# Adaptive Monostatic System for Measuring Microwave Reflections from the Breast

**DOI:** 10.3390/s18051340

**Published:** 2018-04-26

**Authors:** Jeremie Bourqui, Martin Kuhlmann, Douglas J. Kurrant, Benjamin R. Lavoie, Elise C. Fear

**Affiliations:** 1 Schulich School of Engineering, University of Calgary, Calgary, AB T2N 1N4, Canada; djkurran@ucalgary.ca (D.J.K.); brlavoie@ucalgary.ca (B.R.L.); 2 College of Engineering and Architecture of Fribourg, 1700 Fribourg, Switzerland; martin@mkuhlmann.net

**Keywords:** microwave imaging, breast cancer, prototype system

## Abstract

A second-generation monostatic radar system to measure microwave reflections from the human breast is presented and analyzed. The present system can measure the outline of the breast with an accuracy of ±1 mm and precisely place the microwave sensor in an adaptive matter such that microwaves are normally incident on the skin. Microwave reflections are measured between 10 MHz to 12 GHz with sensitivity of 65 to 75 dB below the input power and a total scan time of 30 min for 140 locations. The time domain reflections measured from a volunteer show fidelity above 0.98 for signals in a single scan. Finally, multiple scans of a breast phantoms demonstrate the consistency of the system in terms of recorded reflection, outline measurement, and image reconstruction.

## 1. Introduction

Microwave imaging of biological tissues currently is in development for a range of applications [1,2,3]. Generally, electromagnetic waves with frequency content ranging from 0.1 to 10 GHz are utilized, depending on the intended target. Breast imaging has been the most frequently investigated application, as the breast is easily accessible for imaging, and electrical properties vary between healthy and malignant tissues [4]. Two technology streams have been developed, specifically tomographic and radar-based techniques. The former uses transmitted signals to create an electrical property map of the breast tissue, while radar techniques use reflected signals to create a backscatter intensity map indicating potential breast lesions. This article reports the development of a radar imaging prototype developed to scan breast cancer patients.

While microwave radar breast imaging has been explored for a significant time, only a few systems have been developed for human trials. The University of Bristol has developed a multi-static radar system using a hemispherical array with 31 elements [5], later increased to 60 elements [6]. Their technology was further developed by the Micrima company (Micrima, UK) and currently is in clinical trials [7]. Another hemispherical array was developed at McGill University. This system consists of 16 antennas and was tested on healthy volunteers [8].

At the University of Calgary, we have focused on monostatic systems consisting of a single antenna that scans the circumference of the breast. An initial prototype [9] was developed with the capability to move our sensor in a cylindrical pattern (vertical movement and rotation around a fixed circumference). A point cloud of the outline of the breast was also recorded through a laser measurement system. This prototype was used in a pre-clinical study involving breast cancer patients [10]. This study demonstrated the need for a more adaptable system, given the range of breast sizes and shapes encountered.

As a result of the lack of symmetry of the breast, the distance between the sensor and the breast can vary dramatically. Greater distances significantly limit the amount of energy available to interrogate the breast and deteriorate the signal-to-noise ratio [11]. In addition, as the sensor moves toward the nipple region, the measured signal reflected from the skin has more variation, as the antenna is located further from and not oriented normal to the skin surface. Such variation is detrimental to the efficacy of the algorithm used to reduce the dominant reflection from the skin [12].

This paper presents the next generation of this system, which features improvements to make the system adaptable to different patients and ensure the measurement of a more consistent skin reflection. This requires additional movement capabilities, submillimeter sensor placement accuracy, and an improved laser outline to enable patient-specific scans.

The paper is structured as follows. Section 2 describes the hardware and the scan procedure. Section 3 shows the system performance and validation. Section 4 shows an example of increased consistency of recorded reflections with healthy volunteer data and Section 5 explores the consistency of the system with a simple breast model.

## 2. Prototype System and Scan Procedure

### 2.1. Prototype System and Scan Procedure

**Figure 1:**
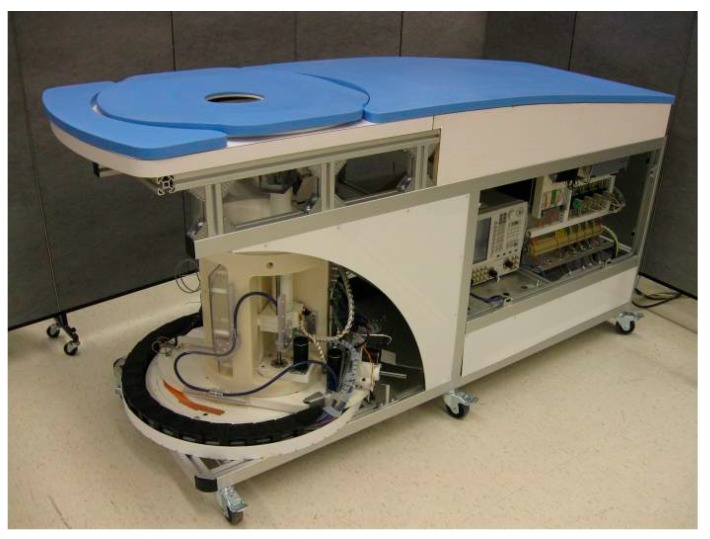
View of the patient compatible monostatic radar system with adaptive sensor placement capability (image source: Sensors 2017, 17, 1658).

The general design of the system, shown in Figure 1, is similar to the one reported in J. Bourqui et al. [9]. It consists of a padded bed on which the woman lies prone. One of her breasts extends through a hole and into the imaging tank located under the bed. The tank contains the microwave sensor [13], a laser sensor to obtain the breast outline, and an arm for sensor repositioning. A camera is positioned on the outside of the tank to monitor the progress during the scan. The tank is filled with canola oil, which acts as a matching medium between the sensor and the breast skin. Canola oil exhibits a relative permittivity of 2.5 and negligible attenuation with conductivity below 0.04 S/m up to 12 GHz.

The principal improvement, compared with the system described in in J. Bourqui et al. [9], resides in the mechanical ability to better position the sensor around the breast. In the previous system, only two degrees of freedom were available. The tank was able to rotate around the breast and the sensor was scanned vertically, creating a cylindrical scan pattern with a fixed diameter.

**Figure 2:**
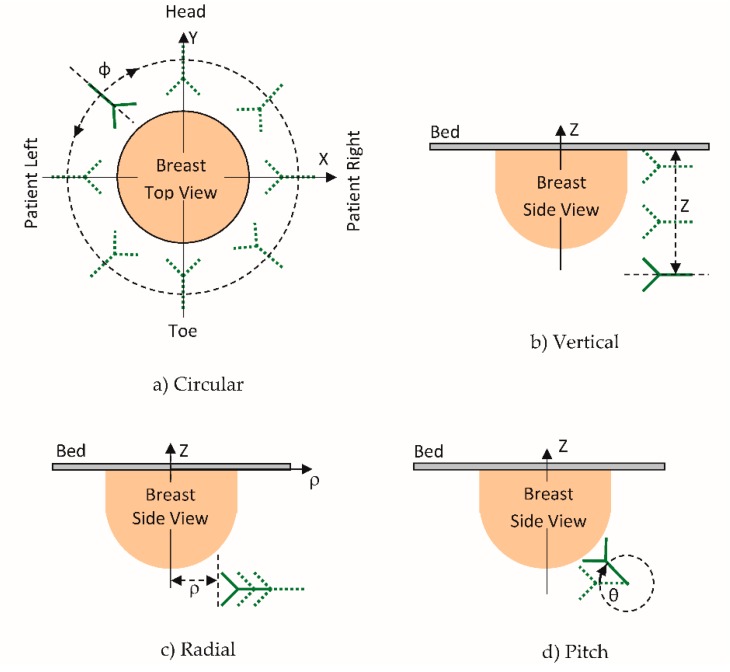
View of the different movement capability: (**a**) circular along Φ allowing rotation over a full revolution around the breast; (**b**) vertical along Z with travel from −20 to −178 mm below the examination bed; (**c**) radial along *ρ*, which can vary from 82 to −28 mm, measured from the sensor tip and the center of the opening, and (**d**) pitch angle, defined by θ, adjustable between 0 and 88°.

**Figure 3:**
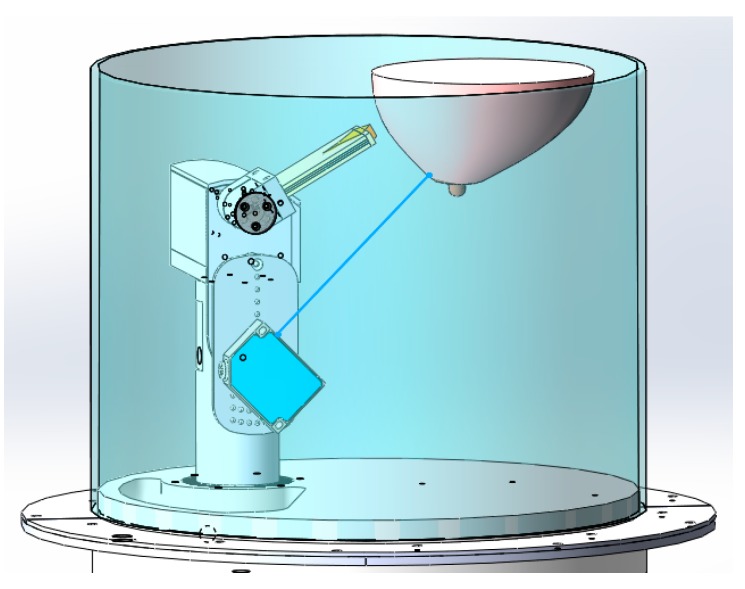
Diagram of the positioning arm in the imaging tank with the antenna and laser attached.

The system reported here introduces two additional movements: radial distance and pitch inclination. Figure 2 provides a graphical description of the four movement capabilities. The sensor can be rotated (Φ) a full revolution around the breast (Figure 2a); the elevation (Z) can be adjusted from −20 mm below the examination bed to −178 mm deep (Figure 2b); the radial distance (*ρ*) between the sensor tip and the center of the opening can vary from 82 to −28 mm (Figure 2c) and the pitch is adjustable between 0 and 88° (Figure 2d). This level of positioning freedom and knowledge of the breast outline enable the sensor placement locations to be patient specific, which improves breast illumination [11]. Figure 3 shows the microwave sensor attached to the positioning arm, along with the laser sensor that detects the outline of the breast. Prototype movements are implemented with brushless DC servo motors including encoders and motion controllers (Faulhaber, Switzerland). Notably the same DC motor (4490H024BS) is used for all axes except for the pitch, which uses a more compact actuator (2444H024BS). The motion controller (MCBL3006S) is used for each axis and communicates with the central computer over dedicated serial communication ports. In comparison to the stepper motors used by the previous system [9], the DC servo motors, in conjunction with the drivers, have the advantage of increased flexibility in terms of acceleration and deceleration. Most importantly, the DC servo motors provide more precise movements, as they are not impacted by problems related to step slipping. The system is entirely controlled by custom LabVIEW (National Instruments) software.

The laser sensor measures the distance to the breast, which is then translated to coordinate values through a linear calibration [14]. The laser outline is first used to verify positioning of the breast. To ensure that the volunteer’s breast is approximately aligned with the rotation center before the scan begins, the outline of the breast is recorded over a full revolution using the circular movement.

The laser outline also provides information on the shape and location of the tissue to enable the design of the patient-specific scan pattern. During a scan, an outline is measured at each azimuth angle (Φ) evaluated by the system. This outline is acquired by moving the positioning arm along the Z-extent at each azimuth position so that the laser records the breast surface from nipple to chest wall. The laser data are continually sampled at a constant rate during the movement and then associated with the spatial location (of the laser sensor) based on the selected movement trajectory.

The trajectory is calculated based on the movement parameters (acceleration, speed, and deceleration) that are given to the motor driver. By starting the movement and the laser acquisition simultaneously, one can associate every recorded laser data point to the laser location in space. Before additional processing, a moving average filter is applied to the recorded laser data to remove any outliers. The breast outline is then generated using the laser data and the laser location as outlined in D. Kurrant et al. [15]. It is critical to have precise control on the acceleration, speed, and deceleration of the actuators to ensure accurate measurement.

The microwave sensor consists of a balanced antipodal Vivaldi antenna with director [13]. This antenna features a directive radiation pattern that focuses the energy toward the breast and ultra-wideband operation (e.g., reflection coefficient below −10 dB from 2.4 to 18 GHz). Note that the prototype can accommodate other antennas if necessary. A cable of length 4.5 m and an RF rotary joint connect the antenna to a vector network analyzer (PNA-L, Agilent Technologies, Palo Alto, CA, USA). To maintain a stable cable response, guiding rails are used to consistently reposition the cable.

Measurements of the reflection coefficient are taken between 10 MHz to 12 GHz at 1200 points with a port power of 10 dBm. As a result of cable loss, this translates to a power level between 7 and −3 dBm at the antenna feed for frequencies between 1 and 12 GHz. To improve sensitivity while keeping the measurement time practical, an intermediate frequency (IF) bandwidth of 1 kHz and an averaging factor of 3 are used. This translates to a measurement time of about 5 s per antenna position with sensitivity of −100 dB for consecutive reflection measurements of a broadband load.

### 2.2. Scan Procedure

During a scan of a breast model or volunteer, the procedure is as follows. Using the circumferential outline acquired with the laser, the operator ensures that the breast is reasonably centered. The scan pattern is then designed in terms of number of azimuth locations and the number of antenna locations at each azimuth.

**Figure 4:**
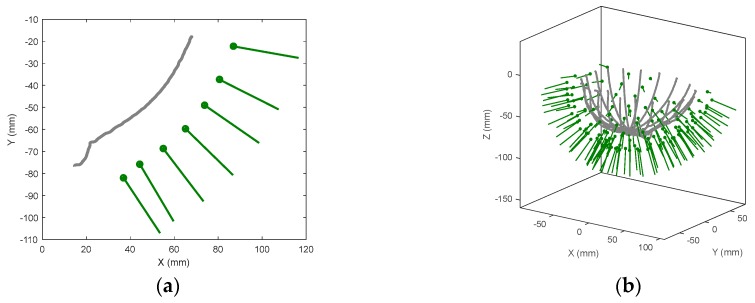
Laser outline for (**a**) a single azimuth; and (**b**) an entire scan of a volunteer breast and the calculated antenna locations shown as line and dots representing the antenna orientation and aperture location respectively.

The outline is first measured at a selected azimuth angle. Next, this recorded profile is used to calculate the locations for the antenna such that it is positioned normal to the skin and at a fixed distance away from the breast [16]. An example of the antenna locations calculated based on an outline is shown in Figure 4a. In addition, a stagger consisting of offsetting the azimuth location is used, e.g., a stagger of 3 adds an azimuth offset for every second and third antenna location. Effectively, the introduction of the stagger improves the illumination coverage over the breast surface. The antenna is moved to each location and the reflection coefficient is measured. A still photograph is also saved for each measurement location using the on-board camera. The images are only used for the post-scan evaluation of the antenna positions. Although these images are very useful at this stage of testing of the technology, they are not required for prototype operation or image formation. The antenna is then repositioned at the next azimuth location and this process is repeated until the entire breast circumference is scanned.

Scan patterns are typically comprised of 20 azimuth positions with seven antenna locations per azimuth, for a total of 140 antenna positions (Figure 4b). This provides a degree of spatial sampling that balances the requirement of providing adequate coverage over a wide range of breast sizes with constraints on the duration of measurement time. Typical total scan time is 30 min, which is in line with modalities such as MRI. After the scan of the breast model or volunteer is complete, an identical scan is performed with only the tank filled with canola oil present (referred as the ‘empty tank’) for calibration purposes. During this scan, an additional tank, also filled with canola oil, is placed above the prototype lid. As the antenna positions tend to be oriented towards the hole in the bed of the prototype (Figure 4a), the additional tank is required to minimize the reflection from the interface between oil and air. The tank is acrylic, which exhibits very similar electrical properties to canola oil. This additional tank has a recessed bottom that precisely fits the opening of the original lid so that no air interface exists.

## 3. System Performance and Validation

System performance, in terms of position of sensors, the accuracy of laser measurements, and microwave measurement sensitivity, is defined and evaluated.

### 3.1. Positioning Performance

One of the key aspects of system performance is the placement of the sensor at a given location with an adequate precision (repeatability) and accuracy. Accuracy is needed as the antenna position is used in the reconstruction algorithm and errors may introduce artifacts in the image. Positioning precision ensures that the measured antenna response remains constant between the actual scan (when the patient is present) and the calibration scan (empty tank). Any difference in position between the two scans will slightly change the cable response and the location of the environment (tank boundaries) relative to the antenna, which affect the overall antenna response.

We explore the impact of positioning errors on images using simulations. For each antenna position, we introduce randomly distributed perturbations with mean of 0 and standard deviation of 2 units to each of the 4 degrees of freedom. Even with this significant perturbation, the images were virtually visually indistinguishable, had no displacement of the main response centroid, and the difference in the overall backscatter response measured using the Modified Hausdorff Distance (MHD) was 0.28 mm [17]. With systematic shifts in antenna positions, similar consistency in images was also noted.

In terms of precision, measurements show that a 0.5 mm change in antenna vertical position can produce a perturbation magnitude on the order of −30 to −40 dB in the reflection coefficient. To obtain a comfortable margin, the system was designed for accuracy and precision of ±0.1 mm and ±0.1° for linear and angular movements, respectively. These values are also in line with realistic performance for a basic positioning system.

To validate the positioning performance, each axis is evaluated individually by commanding movement to a specific position and measuring the actual axis position with an independent measurement device. The circular movement is measured through a graduated wheel (built within the prototype) enabling a resolution of 0.05°. For the radial movement, a digital caliper (0.01 mm resolution) is connected horizontally between the antenna attachment and a stationary point on the examination bed. The same technique is used for the vertical movement with the caliper oriented vertically. For the pitch movement, a digital level is attached to the pitch mechanism. The digital level has a resolution of 0.1°; however, its accuracy degrades to ±0.2° between 10 to 80°. While this exceeds our accuracy goal, it is the best measurement device found for this application.

Testing consisted of moving to approximately 30 locations over the entire travel range. Back and forth movement was included to exacerbate the cumulative effect of residual uncompensated mechanical play in the mechanisms. This test was repeated five times for each axis, resulting in a total of about 150 test locations for each axes. Table 1 shows the maximum and minimum deviation value seen in all the data, as well as the standard deviation (SD).

Overall, the positioning performance was well within the target except for a few locations where the accuracy of circular and pitch movements that were greater than specifications. The maximum pitch error could be attributable to the limited accuracy of the digital level.

### 3.2. Laser Performance

The laser (ILD1300-200 Micro-Epsilon, Ortenburg, Germany) provids a resolution of 0.2 mm for dynamic measurement and a linearity of 0.4 mm. The changing angle of the breast, with respect to the laser beam, also influences the measurement accuracy. Another potential source of error may result from the mechanical positioning. As described in Section 2.1, the distance measured by the laser is used in conjunction with the knowledge of the sensor movement. Any error between the calculated and actual sensor position versus time introduces error into the measured outline.

**Figure 5:**
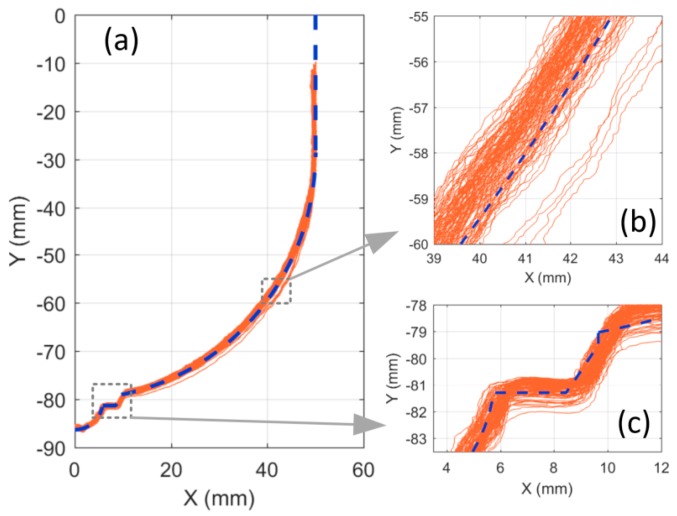
Recorded laser outlines at 120 different azimuths compared to the known breast model outline: overall outline (**a**), close up on the vertical section (**b**), and nipple detail (**c**).

In consideration of these various sources of error, practical measurements are necessary to infer the recorded outline accuracy. To this end, an axially symmetric breast model [18] with known dimensions was scanned. Figure 5 shows the outlines taken at 120 different azimuth angles (every 3°). One observes four curves deviating from the others, most clearly in Figure 5b. These result from the arm movement starting slightly later than the laser recording, which creates an inconsistency between the calculated versus actual laser location. The source of this delay was related to a delay in port communications or background task from the computer. It is important to point out that such delays do not occur frequently (4 out of 120 in this case) and a maximum offset of 1.4 mm was observed from the other profiles. Ignoring these outliers, a maximum variation of ±0.8 mm was measured between all outlines. Deviation from the true profile was approximately ±1 mm which is considered as the accuracy of this scanning technique. This level of accuracy was also shown in a separate study [15].

### 3.3. Microwave Measurement Sensitivity

Microwave imaging systems must measure weak signals reflected by the breast interior. The data used for imaging result from a subtraction of two successive scans: one with the volunteer present and one with an empty (oil-filled) tank. Accordingly, measurement sensitivity is defined as the smallest signal that could be recovered from the difference of two reflection coefficients measured independently. In this situation, the sensitivity is directly related to the overall stability of the measurement system. As analyzed in J. Bourqui et al. [9], the measurement equipment’s intrinsic stability (trace noise), temperature changes over time, and cable movements are influencing factors.

Ensuring measurement stability begins with repeatability of antenna positioning and corresponding cable shape, which are achieved with high positioning precision and cable-guiding rails. However, the most significant sensitivity limitation results from the stability of the measured interaction between the sensor and the imaging tank. There are several physical factors which influence this interaction stability. As a low-loss immersion medium is used, any changes in the tank, such as liquid level variation or appearance of air bubbles, could introduce artifacts. The previous system [9] used a recessed lid structure to maintain a constant oil level in the vicinity of the antenna. Such a system was not possible to implement in this design due to mechanical constraints. Therefore, a pump is used to keep the oil level in contact with the surface of the lid as much as possible and to avoid the formation of air bubbles.

**Figure 6:**
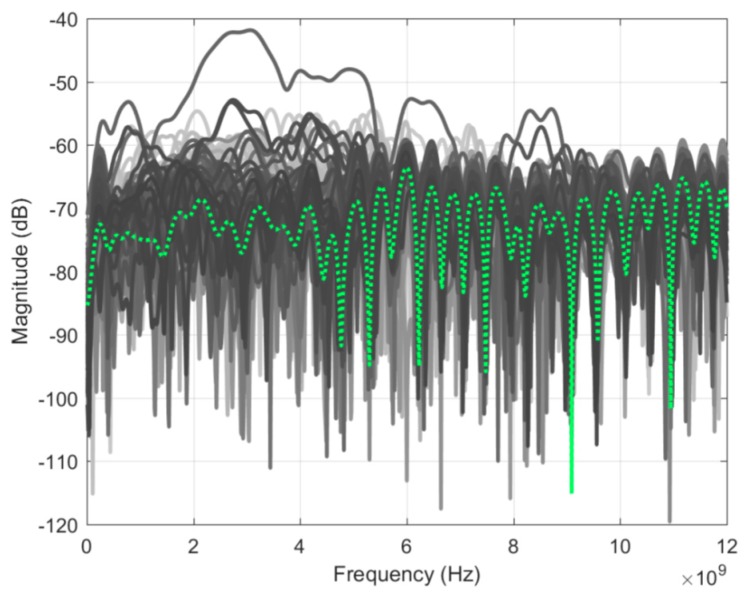
Residual noise for 140 antenna positions after the subtraction of two identical scans of the empty tank. Average value in dotted green.

To assess measurement sensitivity, two identical scans of an empty tank were taken. Figure 6 shows the result of the subtraction of the two scans for all signals along with their average value. Looking at the average, the residual signals were between −65 to −75 dB, which defines the sensitivity of this system. In comparison to the prototype in J. Bourqui et al. [9], the sensitivity is reduced by approximately 5 dB. The decrease is the result of the additional movement complexity, which increases the variability of the background reflection of the tank. From Figure 6, one can observe several outliers, including one close to −40 dB. This typically occurs when an air bubble forms on top of the imaging tank. This does not occur systematically as the pump keeps the oil level reasonably constant and usually prevents the formation of any air bubbles.

## 4. Skin Reflection Stability

A key challenge in microwave breast imaging with a monostatic radar system is the reduction of the dominant reflection from the skin layer. Patient-specific scan patterns provide similar responses from the skin, which are important for effective performance of signal processing algorithms to reduce this reflection [12]. To evaluate this improvement, multiple volunteer scans were analyzed.

**Figure 7:**
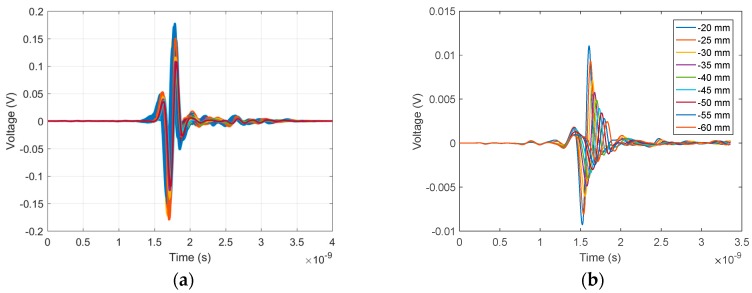
(**a**) Overlay of all 140 skin responses recorded during a volunteer scan. (**b**) Overlay of nine skin responses recorded during a volunteer with the previous prototype. All skin responses in (**b**) are for the same azimuth but at different depth from the chest wall to the nipple.

Figure 7a shows 140 time-domain signals of the skin reflection recorded during a volunteer scan. Significant similarity was observed both in amplitude and waveform for all signals. In comparison, skin reflections recorded along one azimuth location using the previous cylindrical scanning system [9] are shown in Figure 7b. As expected, significant changes in time shift, waveform, and magnitude were observed, demonstrating the significant improvement of adaptive antenna placement.

**Figure 8:**
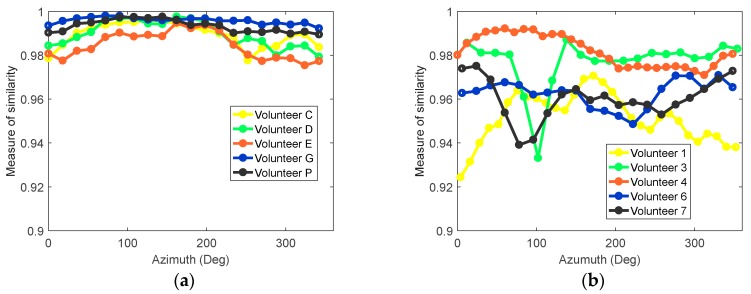
(**a**) Similarity measure of the skin response around the breast circumference for five different volunteers; (**b**) Similarity measure of the skin response around the breast circumference for five different volunteers from the previous prototype.

To further assess the similarity of the response waveform over an entire scan, the cross-correlation between signals was calculated. For a selected antenna at each azimuth location around the breast, a group of neighboring antennas located within a 35 mm radius was identified. The similarity between the skin reflection at the selected antenna and each neighbor was calculated, and the average similarity was calculated. More details on the technique can be found in D. Kurrant et al. [11]. This procedure was applied to five volunteer scans collected using both prototypes; results are shown in Figure 8. With the patient-specific scan, similarity between signals was typically above 0.98 (Figure 8a). The similarity for the cylindrical scanning system is shown in Figure 8b and indicates significantly lower similarity and less consistency between volunteers. This lack of similarity has a negative impact on the performance of the skin subtraction algorithm. The improvements observed with multiple scans of different volunteers demonstrate the advantage of the adaptive antenna placement.

## 5. Imaging of Simple Breast Model

The antenna position, laser outline, microwave measurements, and skin response stability all contribute to accurate imaging. To test these parameters, a simple breast model was scanned multiple times. Consistency of the recorded waveform and laser surface were evaluated and reconstructed images were compared.

The breast model was composed of a 2 mm thick, conically shaped, skin shell as described in J. Garrett et al. [19]. The skin shell was filled with canola oil and a 16-mm diameter tumor-mimicking sphere was placed within the breast model. The corresponding electrical properties of the breast phantom between 1 to 10 GHz are listed in Table 2. More detailed properties can be found in in J. Garrett et al. [19]. Simulations indicate that this imaging scenario requires a system sensitivity of −40 to −60 dB to detect tumor signals, which is within the capacity of the system as it has a sensitivity of −65 to −75 dB.

**Figure 9:**
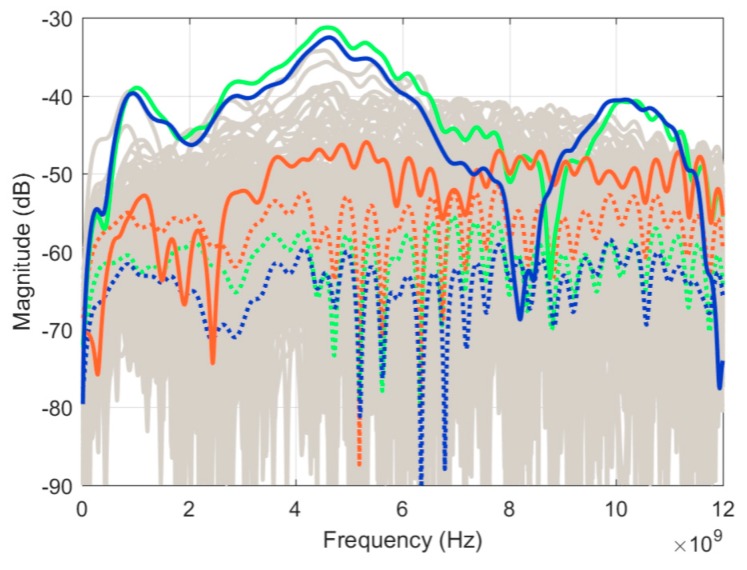
Differences between scans for each signal in the frequency domain. All signals are in grey with average values in dotted green, red, and blue for Scan2-Scan1, Scan 3-Scan1 and Scan3-Scan2 respectively. Residual response at position 127 in solid green, red, and blue for Scan2-Scan1, Scan 3-Scan1 and Scan3-Scan2, respectively.

To assess the consistency of the imaging system, the breast phantom was positioned in the scanner and independently scanned three consecutive times with the same antenna positions. The laser outline was also recorded for each scan. After the three scans, the breast phantom was removed and one scan of the empty tank was performed for calibration. Figure 9 shows the differences (between scan pairs) for all signals collected during each scan, leaving only the residual (unwanted) reflections. All the curves (3 × 140 = 420) are displayed in the same shade of grey. Since single curves could not be distinguished, the relevance of this graph is the extent of variation of the residuals. The average for each scan pair is displayed with a dotted colored curve, while the residual at selected positions are highlighted in solid colored lines; these will be discussed in the next paragraph. Ideally, the average magnitude should be similar to the residuals recorded for the empty tank in Figure 6. However, higher values were observed by about 10 dB. This is explained as each measured signal includes the skin layer reflections, which are significantly higher magnitude than the empty tank reflections. Therefore, the detrimental effect of the cable response variation (especially phase response) is accentuated as a result of the higher overall reflection. Second, slight variations in the antenna positions between scans (with respect to the skin) have a greater effect on the recorded signal compared to an empty tank measurement.

**Figure 10:**
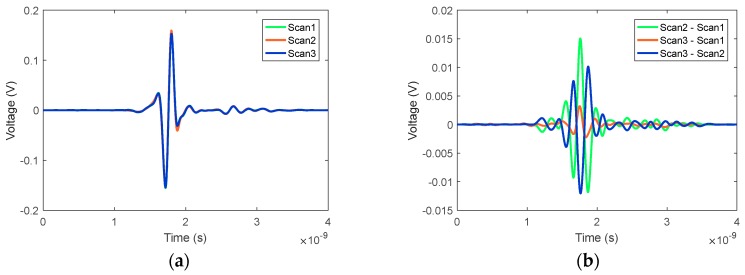
Calibrated (antenna response removed) signals recorded at the same location (position 127) for the three consecutive scans, showing the highest residual difference between scans. (**a**) Time domain signals for the three scans and (**b**) differences between scans.

Figure 9 also shows that several signals were outliers from the average. These signals were recorded close to the measurement lid where any air bubbles could significantly influence the signal. One of these signals, recorded at position 127, is highlighted in Figure 9 for all three scan pairs and represented in the time domain in Figure 10. In Figure 10a, the reflection from the breast model skin layer and its late time response can be observed in the original signals. Differences in the main skin response are clearly visible, while the subtraction of the signals in Figure 10b provides the details. As the residual between Scan3 and Scan1 was small, one may conclude that the signal recorded during Scan2 was the outlier. Inspecting the images taken by the onboard camera while the measurements were recorded, a large air bubble was present at that specific antenna location during Scan2 and not for the other scans. This confirms that air bubbles are a significant limiting factor on the system sensitivity. However, it is important to observe that the effect of this air bubble is predominantly on the dominant part of the skin reflection (between 1.5 and 2 ns), which can potentially be filtered out by the skin subtraction algorithm.

To evaluate the similarity between the recorded profiles, the surface estimation procedure described in D. Kurrant et al. [15] was applied to the measured laser points to reconstruct the three different surfaces. Using Equations (5) and (6) in D. Kurrant et al. [15], the similarity between the three reconstructed surfaces was measured at 0.984, 0.977, and 0.978. These values are comparable to the ideal case when the measured surfaces were compared to the true surfaces [15].

**Figure 11:**
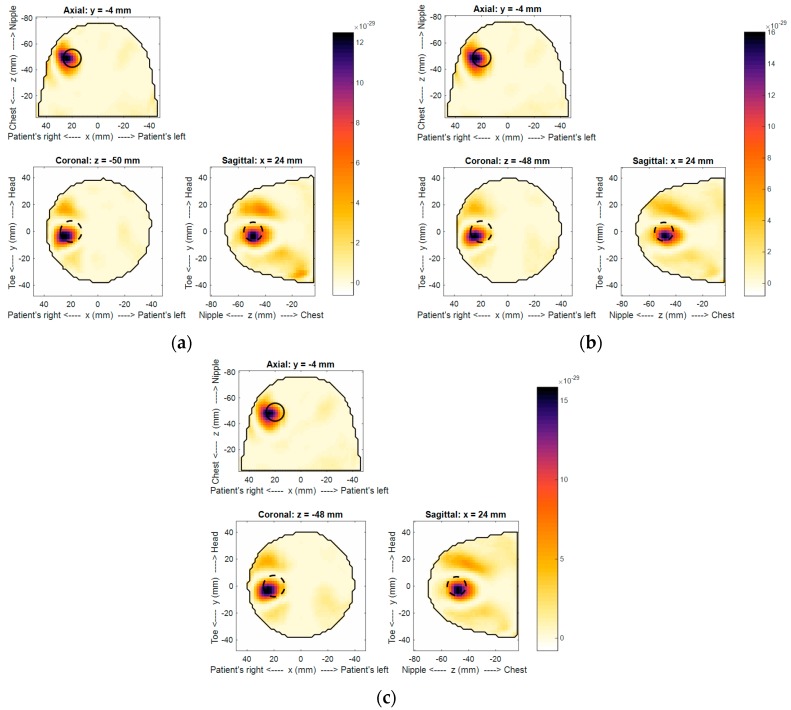
Reconstructed backscatter intensity images of the three consecutive scans (**a**–**c**) of the breast phantom. Dashed circles represent the location of the 16 mm target. Note that the color bar is unitless.

Using the technique described in E.C. Fear et al. [10], images were reconstructed for the three different scans and are shown in Figure 11a–c. Note the image reconstruction, including data loading, takes 37 s on a standard desktop computer. Detection of the tumor was noted in all images, confirming that the target response was within the sensitivity of the system. Only marginal differences were observed among all images, confirming the overall consistency of the system. The target was detected at the expected location. Notably, although best efforts were made to accurately place the target within the phantom, the expected location, noted by the dashed circle in Figure 11, may not be exact. In terms of detected location variation, the change of the centroid location between scans was measured. A maximum of 1.22 mm and minimum of 0.25 mm were measured between scans 1–3 and scans 2–3, respectively. Additionally, the difference in the overall backscatter response measured using the MHD as described in B.R. Lavoie et al. [17] ranged between 0.49 mm and 0.17 mm. The highest and lowest values were between scans 1–3 and 2–3, respectively. This level of consistency confirms the precision of the sensor positioning, laser outline, and microwave reflection measurement as the image reconstruction depends on all of these factors. Artifacts in the images were small and largely related to the echo of tumor response, suggesting effective reduction of the skin reflection, as expected with similar skin responses.

## 6. Conclusions

A prototype system for measuring microwave reflections from the human breast is described and performance evaluated. With four degrees of freedom in sensor positioning and knowledge of the breast shape through laser scanning, the microwave sensor could be placed adaptively around the breast. Scan time was approximately 30 min to acquire microwave reflections between 10 MHz to 12 GHz at 140 antenna locations.

The positioning of the microwave sensor was measured to be within ±0.1 mm and ±0.1° for linear and circular movements, respectively. Using a laser sensor system, the outline was acquired with an accuracy of ±1 mm. The system was determined to be sensitive enough to measure microwave reflections from the breast 65 to 75 dB below the incident signal. This sensitivity level enables the measurement of reflections from tumors in the human breast. However, when the tumor is deep in the glandular tissue, only lower frequency reflections are measurable, which reduces the imaging resolution. For example, with a 10 mm diameter tumor embedded deep in glandular tissues, only signals between 1 and 3 GHz could be measured with this system.

With adaptive sensor placement, measurements on volunteers demonstrated very good stability of the skin reflection waveform, which optimizes the efficacy of the algorithm used to remove this dominant reflection. The overall consistency of the system was tested by scanning a breast phantom three consecutive times, demonstrating very similar results in terms of recorded signals, laser surface measurement, and reconstructed images. The inclusion present in the breast model was localized repeatedly at the expected location with maximum deviation of 1.22 mm. This improved prototype is a significant advancement compared to its previous version; next steps include scanning volunteers with and without breast health issues.

## Figures and Tables

**Table 1:** Maximum, minimum, and standard deviation of positioning error observed among all the position tested.

Movement	Precision			Accuracy			Unit
	Max	Min	SD	Max	Min	SD	
Circular	0.04	−0.05	0.01	0.1	−0.11	0.04	deg
Radial	0.02	−0.05	0.005	0.08	−0.07	0.03	mm
Vertical	0.02	−0.01	0.01	0.05	−0.04	0.02	mm
Pitch	0.08	−0.08	0.03	0.20	−0.20	0.10	deg

**Table 2:** Electrical properties of the simple breast model elements.

Object	Relative Permittivity (1)	Conductivity (S/m)
Skin	30 to 20	1 to 2.5
Canola Oil	2.5	0 to 0.04
Tumor	60 to 30	7 to 15
